# Sound pressure level spectrum analysis by combination of 4D PTV and ANFIS method around automotive side-view mirror models

**DOI:** 10.1038/s41598-021-90734-1

**Published:** 2021-05-27

**Authors:** Dong Kim, Arman Safdari, Kyung Chun Kim

**Affiliations:** 1 Rolls-Royce University Technology Centre, Pusan National University, Busan, 46241 Republic of Korea; 2 Department for Management of Science and Technology Development, Ton Duc Thang University, Ho Chi Minh City, Vietnam; 3 School of Mechanical Engineering, Pusan National University, Busan, 46241 Republic of Korea

**Keywords:** Engineering, Optics and photonics

## Abstract

This paper proposes a data augmentation method based on artificial intelligence (AI) to obtain sound level spectrum as predicting the spatial and temporal data of time-resolved three-dimensional Particle Tracking Velocimetry (4D PTV) data. A 4D PTV has used to measure flow characteristics of three side mirror models adopting the Shake-The-Box (STB) algorithm with four high-speed cameras on a robotic arm for measuring industrial scale. Helium filled soap bubbles are used as tracers in the wind tunnel experiment to characterize flow structures around automobile side mirror models. Full volumetric velocity fields and evolution of vortex structures are obtained and analyzed. Instantaneous pressure fields are deduced by solving a Poisson equation based on the 4D PTV data. To predict spatial and temporal data of velocity field, artificial intelligence (AI)-based data prediction method has applied. Adaptive Neural Fuzzy Inference System (ANFIS) based machine learning algorithm works well to find 4D missing data behind the automobile side mirror model. Using the ANFIS model, power spectrum of velocity fluctuations and sound level spectrum of pressure fluctuations are successfully obtained to assess flow and noise characteristics of three different side mirror models.

## Introduction

Most drivers know that they must turn up the volume to listen to their favorite radio channels on the highway and speak louder to talk to the passenger. This is a direct result of the turbulence that induced pressure fluctuation around window. The pressure fluctuation creates vibrations of window glass plates, which generate some of the internal noise through the air inside the vehicle. There are five types of wind noise sources: turbulent boundary layers, separated and reattaching flow, cavity flow, vortex shedding, and leak (or aspiration) noise^1^. Side view mirrors can contribute to significant interior noise in the automobile cabins. The vortices induced by the mirrors produce powerful exterior noise and hydrodynamic impingement, which excite the downstream windows. The interior noise can be reduced by suppressing the turbulent flow separation on the mirrors.

To measure noise sources, wind tunnel testing is still a common method in the automotive industry^1–8^. Various turbulent structures around exterior mounted vehicle mirrors have been investigated experimentally by Rinoshika et al.^9^. In addition, Khalighi et al.^10^ conducted Particle Image Velocimetry (PIV) and pressure measurements behind the two outer side view mirrors of the vehicle in the wake region. Kim et al.^7^ measured surface flow and wake structure of passenger car side view mirror. In order to measure velocity and pressure which are important properties for determining aerodynamic performance, point measurement methods such as a pressure transducer and a hot-wire are adopted or 2D planar PIV measurement is being conducted. In the case of pressure measurement, it cannot identify the nature of the noise mechanism as well as time and effort, as the microphone arrangement is installed to measure floating noise and to use a trial-and-error method to modify features. Far field data using microphone arrays do not provide enough information on the source mechanism, so it relies on a numerical analysis or source model to understand additional information from the source region. In addition, most PIV measurements are limited to 2D. Obtaining pressure field after performing 3D flow analysis by direct numerical simulation (DNS) or large eddy simulation (LES) is difficult to apply to a complex shape or a high Reynolds number due to computational cost and turbulence model. In addition, the results of the numerical analysis must be verified by experiment.

Today, pressure from PIV techniques provide an alternative to the complex devices of a wind tunnel model with pressure tap for pressure measurement^11–13^. Once velocity material derivatives is accurately calculated, the established time-resolved pressure from PIV procedure solves the incompressible Poisson equation for pressure. Due to uncorrelated random errors in consecutive PIV snapshots, recent studies have employed a Lagrangian tracking^14^ approach to obtain the velocity material derivative from a series of consecutive time-resolved velocity measurements. The combination of tracer particle technology for large-scale wind tunnel experiments (helium filled soap bubbles (HFSB)) and coaxial volumetric velocimetry with the advancements in 3D particle motion analysis (Shake-the-Box) has shown the potential to evaluate the velocity and pressure field in volumes of several liters^15^. An accurate determination of the velocity material derivative in turbulent flows requires full 3D evaluation of the velocity and acceleration field, which is currently possible by high-speed, high-resolved experiment.

Turbulent flow generally involves multidimensional physics, including space and time, which have high dimensions with rotating and transforming intermittent structures. This feature provides an opportunity for the application of artificial intelligence (AI) methods, such as machine learning to predict the modeling and analysis of turbulent flow. Neural networks are popular in a range of areas, including self-driving cars and weather forecasts. Recently, there have been interesting studies on the use of neural networks for fluid dynamics^16–25^, including turbulence modeling^18,21^. The Adaptive Neuro-Fuzzy Inference System (ANFIS) method can learn many physical models and can be very useful in chemical engineering, pharmaceuticals, and industry^26^. This method calculates the changes in linguistic concepts into mathematical or computational ones, which can alter its behavior in different environments and learn to calculate the behavior of many processes that may be uncertain. Neuro-fuzzy computing methods are an example of such techniques. These methods take advantages of machine learning capability of artificial neural networks, and fuzzy inference systems to make reasonable decisions according to the if–then rules^27^.

This paper proposes an AI-based data prediction technique combining the 4D robotic PTV measurement data and an ANFIS framework for fluid dynamic research. The main objectives of this study is intended to establish the ANFIS as an auxiliary tool to collaborate with the 4D PTV approach as well as sound pressure level spectrum analysis. To estimate turbulent pressure field from the time-resolved three-dimensional velocity field data obtained by 4D PTV method, high-resolution of temporal and spatial data should be prepared since second derivatives are included in Navier–Stokes equation. For the purpose, we used a machine learning model. For a good prediction, ground-truth data is necessary. The 4D PTV data include some error vectors, but they are ground truth data obtained by experiments. In addition, as far as the authors aware of, there is no study to use the AI algorithms for developing the sound pressure level spectrum relationship between the experimental results. We use experimental data of fluid flow for different time steps as a data set, and with the ANFIS method. After training all patterns of fluid, the AI predicts missing times with the experimental method, which has not been used in the training method. Learning 3D or 4D flow patterns through AI can result in excellent generalization ability of the resulting model and a decrease in error and noise because it learns statistical data based on the neural network algorithm, which can be stored in a database. The instantaneous pressure field was estimated by solving the Poisson equation using 4D PTV data. The ANFIS model was trained using the time-resolved three-dimensional PTV data measured in the wake region of the side-view mirror model for automobiles as a ground truth. To determine the three-dimensional flow structure and noise characteristics of the side-view mirror model, the instantaneous velocity and pressure field were deduced from the ANFIS model. Vortex shedding phenomena and flow-induced noise are discussed based on the power spectrum and sound pressure level spectra.

## Methods

### 4D Lagrangian robotic PTV measurements of side-view mirror models

The side-view mirror of a vehicle should be designed with a shape that minimizes the aerodynamic noise and aerodynamic resistance^28^. Previous studies examined the vortex characteristics of a vehicle’s side-view mirror using a computational fluid analysis technique or measured the time-average velocity field of a wake flow field using a two-dimensional PIV technique^4^. The flow noise generated in the side-view mirror was measured on the wall surface using a microphone array, but the relationship between the characteristics of the flow structure and noise needs to be investigated^29^. For the optimal design of the automobile side-view mirror, information on the pressure field of flow and the 3D flow field are required.

**Figure 1:**
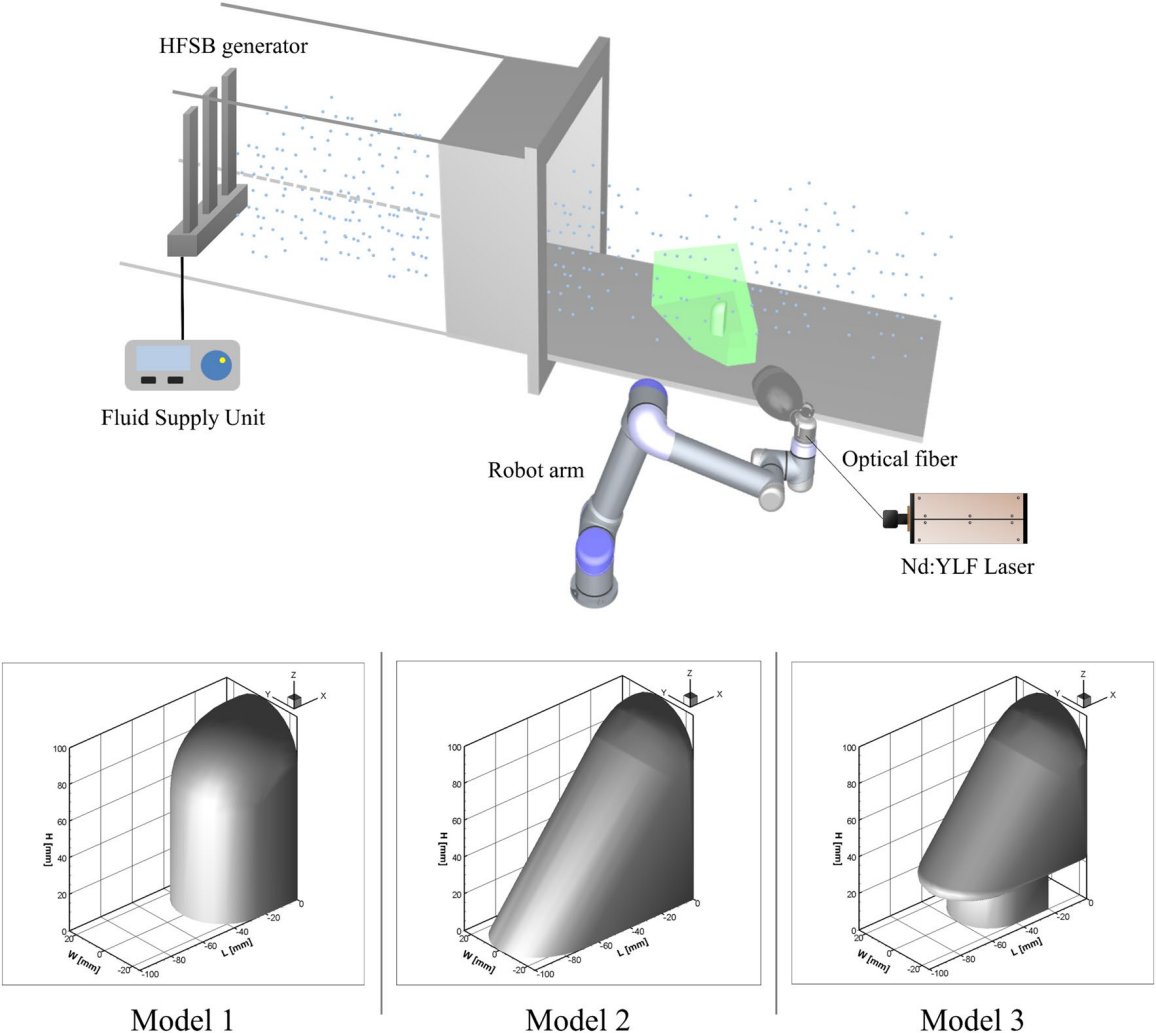
Experimental setup for time-resolved Lagrangian particle tracking velocimetry and side-view mirror models used in this study.

In this study, the time-resolved three-dimensional velocity field of the flow passing through the side-view mirror model was measured using a robotic PTV equipped with four high-speed cameras and the Shake-The-Box (STB) algorithm^30^. To characterize the flow structures around an automotive side-view mirror model, helium-filled soap bubbles were used as tracers in the wind tunnel experiment as shown in Fig. 1. The coaxial volumetric velocimeter (CVV) probe was installed on a collaborative robotic arm UR5 from Universal Robots^31^. The robotic arm provided three rotation and three translation axes movement. The accuracy of the translation was approximately 0.1 mm. The robotic arm’s position could be controlled and simulated using RoboDK software. The light source was a Quantronix Darwin Duo Nd:YLF diode-pumped laser (λ = 527 nm, 2 × 25 mJ pulse energy at 1 kHz). The velocimeter probe consisted of four CMOS cameras equipped with objectives with a 4 mm focal length. The spatial resolution of image sensor is 640 × 452 pix^2^ to 300 × 200 mm^2^. The cameras were integrated within an oval body (LaVision MiniShaker Aero). For volume illumination, the laser light was transmitted with an optical fiber towards the CVV head, where the beam was expanded with a spherical lens^31^. The mirror model has an aspect ratio of H/W = 2. L in the streamwise direction (x-axis), where W is the spanwise direction (y-axis), and H is the direction normal to the wall (z-axis). The experiment was conducted with a free-stream velocity of 13 m/s and a Reynolds number of 85,752. Table 1 lists the experimental conditions.

**Table 1:** Experimental conditions for the 4D PTV measurements.

Velocity of free-stream	13 m/s
Reynolds number	85,752
Boundary layer thickness at x/H = − 1	$\delta /H \approx 0.2$
Height of model (H)	10 cm
Aspect ratio of the model	H/W = 2
Streamlined curvatures of the model	R/H = 0.4 (top) R/H = 0.25 (front)
Image acquisition frequency	867 Hz
Spread angle of illumination	40^o^
Blockage ratio	1.4%

The measurements were conducted in an open jet open-return-circuit wind tunnel with a square contraction ratio of 1:4 and a 60 × 60 cm^2^ cross-section. The velocity profile was measured in the free-stream region using 2D time-resolved PIV. The side-view mirror model was immersed in the turbulent boundary layer in the free-stream region with respect to the model height at δ/H ≈ 0.2. The field of view was checked using the reflectance produced by the laser beams. The probe was repositioned if the reflection exceeded 256 counts. If both the field of view and reflections were acceptable, the viewing information could be stored in a Davis 10 program. A Fluid Supply Unit was operated to seed with the tracer particles, and the seeding concentration was evaluated by a visual inspection before the data are acquired. Once the seeding concentration was satisfactory, the data were acquired (20,000 images at 867 Hz).

**Figure 2:**
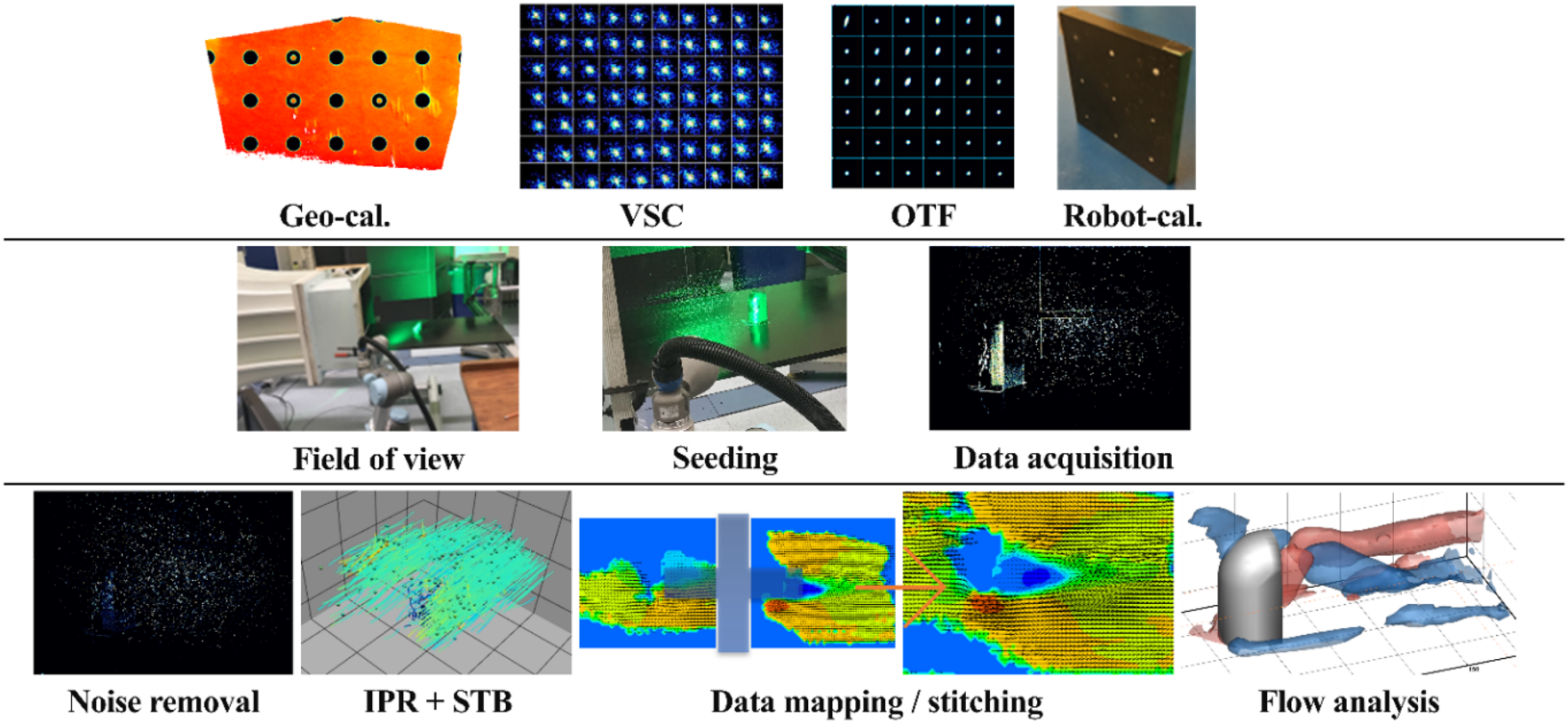
Schematic diagram of data acquisition using the robotic 4D PTV; 1st row represents calibration process; 2nd row represents data recording process; 3rd row represents data reduction and post-processing.

Figure 2 presents a schematic diagram of the working principles of robotic volumetric PTV schematically. The calibration process included the following: geometrical calibration^32^, volume self-calibration^33^, optical transfer function^34^, and robot calibration^31^. After calibration, the robotic arm was positioned to define the field of view and move the velocimeter to the desired position via a robot-control tablet. Post-processing for data reduction is summarized as follows. Raw images were pre-processed using Butterworth filters^35^ to eliminate the background noise and reflection. Pre-processed images were provided as an input to the STB algorithm^14^ to reconstruct the particle track. The data from the camera reference system XYZ_camera_ were converted to the global reference system XYZ_global_, which was aligned with the free stream. The representation of Lagrangian tracking was then mapped to a structured grid using a binning process. The grid elements of the interrogation volume or bin were a 20 × 20 × 20-mm^3^ cube with 75% overlapping, and a velocity vector field with a 5-mm vector pitch was produced. The maximum uncertainty of the in-plane position given by the standard deviation of x and y is estimated on the order of 0.5 mm, while the value for the depth position is 2.5 mm, as expected, according to the small tomographic aperture of the camera system.

### Pressure field evaluation from 4D PTV data

Instantaneous pressure, *p*, can be calculated by solving the Poisson equation^11^.

1 $$ \nabla^{2} p = \nabla \cdot \left( { - \rho \frac{{D{\varvec{u}}}}{Dt} + \mu \nabla^{2} {\varvec{u}}} \right) $$

with the von Neumann boundary conditions on all volume boundaries except for the top side. At the top side, a Dirichlet boundary condition is specified from the Bernoulli equation. At the boundaries, the von Neumann condition was applied, as proposed by Ebbers and Farnebäck^12^. The application of the von Neumann boundary condition will yield the solution of the pressure field up to a finite integration constant. To eliminate the latter, the Dirichlet condition needs to be specified at a known reference location. For the present data, the pressure far upstream of the test object was matched to the expected free-stream pressure. Visualization of the vorticity distribution confirmed the irrotational flow at the top boundary of the measurement. The material derivative in Eq. (1) was evaluated using the Lagrangian technique^13^.

### Data prediction using Adaptive Neuro-Fuzzy Inference System (ANFIS)

**Figure 3:**
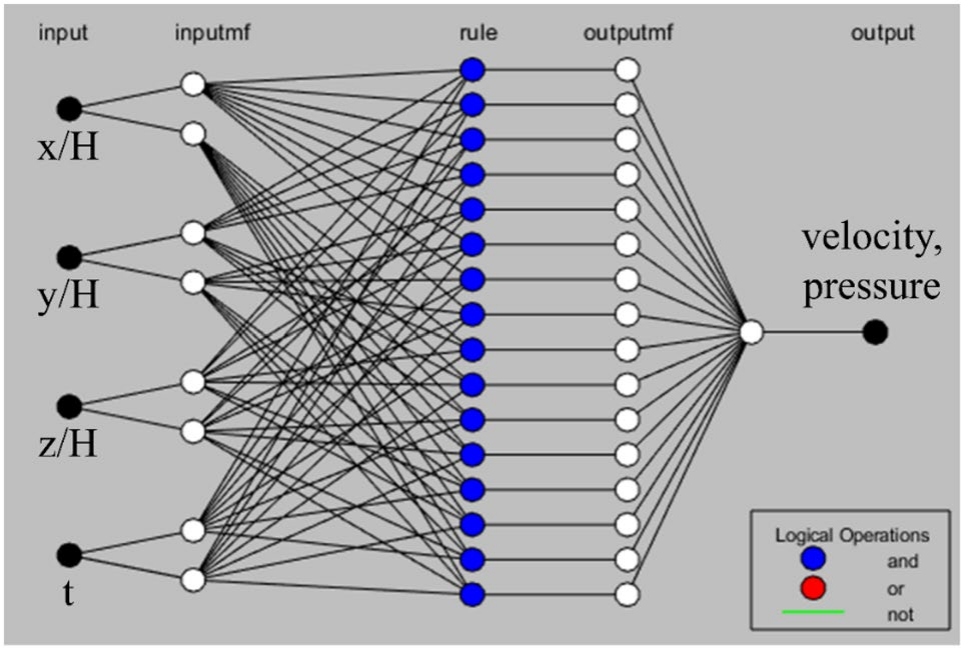
ANFIS structure with a four-input first-order Sugeno fuzzy model in MATLAB 2017a. 4 inputs of coordinates system, and time t were applied and the output was the instantaneous 3D velocity components and pressure.

Figure 3 shows the simple structure of the ANFIS model. The training process can be mathematically regarded as an optimization problem to determine the weighting factor. Given the input data set $x$ and the desired output data set $y$, ANFIS aim to find the optimal weight $w$ in a machine-learned model $F$ that acts as a nonlinear regression function such that $F\left( {x;w} \right)$ $\approx$ $y$. In the present case, $x$ and $F\left( {x;w} \right)$ represent the low-resolution and reconstructed high-resolution data, respectively. The weight $w$ is optimized between the desired high-resolution output $y$ and the ML model output $F\left( {x;w} \right)$ is minimized. The model was used to predict the time-resolved 3D flow characteristics of the side-mirror model with two membership functions as input. Four inputs of the x, y, z coordinates, and time t were applied to obtain the time-resolved three velocity components, and the output was the instantaneous 3D velocity components and pressure. The first-order Sugeno fuzzy model with fuzzy if–then rules were also used. The output of the ith node in layer l is $O_{1,i}$.

Layer 1: Each node i in the first layer is an adaptive node with a node function.

2 $$ \begin{aligned} O_{1,i} & = {\upmu }_{{{\text{Ai}}}} \left( {\text{x}} \right),\;{\text{for}}\,{\text{i}} = 1,{ }2,{ }\;{\text{or}} \\ O_{1,i} & = {\upmu }_{{{\text{Bi}} - 2}} \left( {\text{y}} \right),\;{\text{for }}\,{\text{i}} = 3,{ }4{ }\;{\text{or}} \\ O_{1,i} & = {\upmu }_{{{\text{Ci}} - 4}} \left( z \right),\;{\text{for}}\,{\text{ i}} = 5,{ }6 \\ \end{aligned} $$

where x, y, and z are the inputs to node i, and ${\mathrm{A}}_{\mathrm{i}}$, ${\mathrm{B}}_{\mathrm{i}-2}$, and ${\mathrm{C}}_{\mathrm{i}-4}$ are the associated linguistic labels. ${\mathrm{O}}_{\mathrm{l},\mathrm{i}}$ is any suitable parameterized membership function of a fuzzy set. A fuzzy set is described entirely using its membership function. A generalized bell function was applied because of its great capabilities for the generalization of nonlinear parameters:

3 $$ \mu_{A} \left( x \right) = \frac{1}{{1 + \left( {\frac{{x - c_{i} }}{{a_{i} }}} \right)^{{2b_{i} }} }} $$

where $\left\{ {{\text{a}}_{{\text{i}}} ,{\text{b}}_{{\text{i}}} ,{\text{c}}_{{\text{i}}} } \right\}$ is a variable set. The bell-shaped function differs according to the values of the variables, so it shows various kinds of membership functions for fuzzy set A. The variables in the first layer are known as premise variables.

Layer 2: Each node in the second layer is a fixed one, and its output is the result of all incoming signals:

4 $$ O_{2,i} = w_{i} = \mu_{{{\text{Ai}}}} \left( x \right)\mu_{{{\text{Bi}}}} \left( y \right)\mu_{{{\text{Ci}}}} \left( z \right),{\text{ i}} = 1,{ }2,{ }3 $$

Every node output indicates the firing strength of a rule.

Layer 3: Each node in the third layer is a fixed one. The ith node calculates the proportion of the firing strength of the ith rule in the sum of the firing strength of all rules:

5 $$ {\text{O}}_{{3,{\text{i}}}} = \overline{{w_{i} }} = \frac{{w_{i} }}{{w_{1} + w_{2} }}{ ,}\;{\text{i = 1,2,3}} $$

For convenience, the outputs of this layer are called the normalized firing strengths.

Layer 4: Each node in this fourth layer is an adaptive node with a node function:

6 $$ O_{4,i} = \overline{{w_{i} }} f_{i} = \overline{{w_{i} }} u_{i} = \overline{{w_{i} }} (p_{i} x + q_{i} y + r_{i} z + s_{i} t + h_{i} ) $$

where $\overline{{w_{i} }}$ is the normalized firing strength from the third layer, and $\left\{ {{\text{p}}_{{\text{i}}} ,{\text{q}}_{{\text{i}}} ,{\text{r}}_{{\text{i}}} ,s_{i} } \right\}$ is node’s variable set. In this layer, the variables are referred to as consequent parameters.

Layer 5: In the fifth layer, the single node is a fixed node that calculates the total output as the summation of all incoming signals:

7 $$ O_{5,i} = \mathop \sum \limits_{i} \overline{{w_{i} }} u_{i} = \mathop \sum \limits_{i} w_{i} u_{i} /\mathop \sum \limits_{i} w_{i} $$

Different variables in the ANFIS structures were identified using a hybrid learning method. In the forward pass, functional signals moved forward until they reached Layer 4. The consequent variables were identified by the least-squares estimate. In the backward pass, the error rates moved backward. The gradient descent updates the premise parameters.

The accuracy and performance of the ANFIS method were evaluated based on the statistical parameters. The root mean square error (RMSE) was used to compare the difference between the ANFIS prediction values and the measured data:

8 $$ {\text{RMSE}} = \sqrt {\frac{{\mathop \sum \nolimits_{i = 1}^{n} \left( {O_{i} - P_{i} } \right)^{2} }}{n}} $$

where O, P, and n are the measured data, predicted data, and number of data, respectively. The correlation coefficient (R^2^) is a criterion that illustrates how well the ANFIS data fit the measured data.

9 $$ R^{2} = {{\left[ {\mathop \sum \limits_{i = 1}^{n} \left( {O_{i} - \overline{{O_{i} }} } \right)\left( {P_{i} - \overline{{P_{i} }} } \right)} \right]2} \mathord{\left/ {\vphantom {{\left[ {\mathop \sum \limits_{i = 1}^{n} \left( {O_{i} - \overline{{O_{i} }} } \right)\left( {P_{i} - \overline{{P_{i} }} } \right)} \right]2} {{ }\mathop \sum \limits_{i = 1}^{n} \left( {O_{i} - \overline{{O_{i} }} } \right)\mathop \sum \limits_{i = 1}^{n} \left( {P_{i} - \overline{{P_{i} }} } \right)}}} \right. \kern-\nulldelimiterspace} {{ }\mathop \sum \limits_{i = 1}^{n} \left( {O_{i} - \overline{{O_{i} }} } \right)\mathop \sum \limits_{i = 1}^{n} \left( {P_{i} - \overline{{P_{i} }} } \right)}} $$

**Figure 4:**
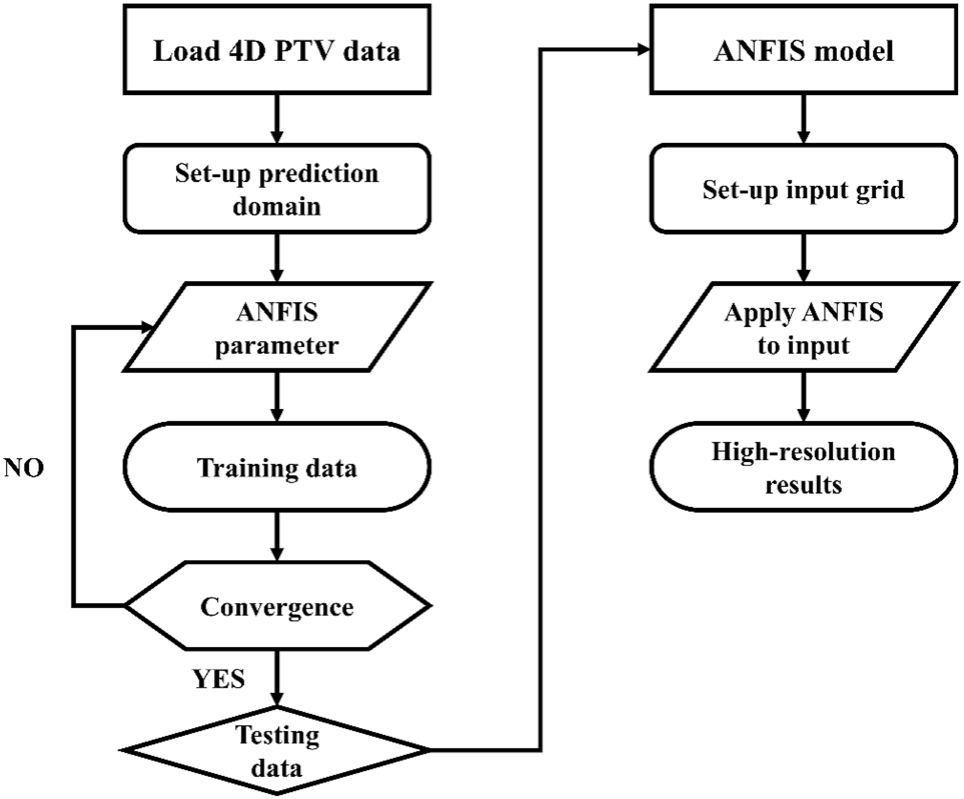
Flow chart of the ANFIS prediction and data prediction of 4D PTV data. The values of the convergence criteria were based on R^2^ > 0.99 and RMSE < 0.01.

**Figure 5:**
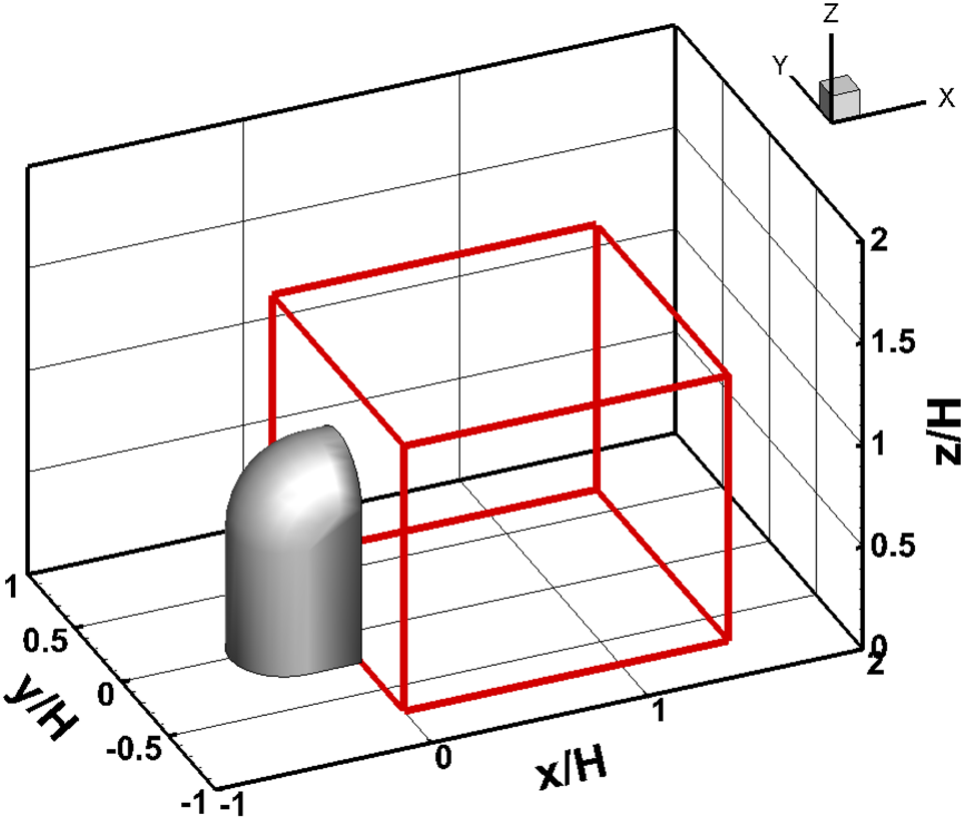
Training domain for ANFIS prediction (red box). The selected domain was used to observe the flow characteristics of the wake region with ranges of 0 < x/H < 1.5, − 0.7 < y/H < 0.7, and 0 < z/H < 1.3.

Figure 4 presents a flow chart of the ANFIS prediction process. The first step was to load the measured 4D PTV data and set the domain of the desired area. In Fig. 5, the red box shows the ANFIS prediction domain. The selected domain was used to observe the flow characteristics of the wake region with ranges of 0 < x/H < 1.5, − 0.7 < y/H < 0.7, and 0 < z/H < 1.3. Subsequently, the ANFIS parameters were applied to train the AI. The ANFIS generation parameters include the percentage of the training and testing data, number of membership functions, type of input membership function, and type of output membership function. To train the ANFIS structure, the parameters included the number of epochs, error goal, initial step size, and the decrease and increase rates of the step size. After setting up the ANFIS parameters, ANFIS training can be started using the measured data, and the convergence can be checked. The values of the convergence criteria were based on R^2^ > 0.99 and RMSE < 0.01. If the values of the convergence criteria are satisfactory, the obtained ANFIS results were applied to the testing data. After checking the convergence of the test data, the ANFIS model was used to predict the data of the 4D PTV data. A good ANFIS model can make entirely new predictions without data in the training process. When a new ANFIS mesh domain is created, the required results can be predicted using the developed ANFIS model. The training cases were classified into three main categories based on the input membership function, percentage of training and testing data, and number of epochs.

Table 2 lists the ANFIS parameters for the input membership function. The experimental results on velocity components and pressure of the side mirror model were used for the sensitivity and accuracy test of the ANFIS model. The numbers of nodes of x, y, and z in the domain were the same as those of the experimental velocity field. On the other hand, 6,666 images (every fourth time-resolved data (289 Hz) were used for training because of the memory limit of the computer. The maximum number of input nodes was 145,585,440. In this case, 70% of the experiment results were used as input to the ANFIS for training. The remaining data were used as testing data to check the prediction results. The convergence was checked while increasing the number of input membership functions. The first three cases had two, three, and four input membership functions with 1000 epochs. After checking how many epochs were satisfactory and converged, the last three cases were examined with five, six, and seven input membership functions, and the epochs were reduced to 300.

**Table 2:** ANFIS parameters used for convergence and sensitivity test.

Parameters	Input
Input	28 × 30 × 26 × 6,666 = 145,585,440 nodes (x/H, y/H, z/H, t)
Target	u, v, w, p
Percentage of training and testing data	Training (70%) Testing (30%)
Type of input membership function	Generalized bell function
Number of input membership functions	2, 3, 4, 5, 6, 7
Number of fuzzy rules	16, 81, 256, 625, 1296, 2401
Epochs	1000 (The number of input membership function = 2, 3, 4) 300 (The number of input membership function = 4, 5, 6)

**Figure 6:**
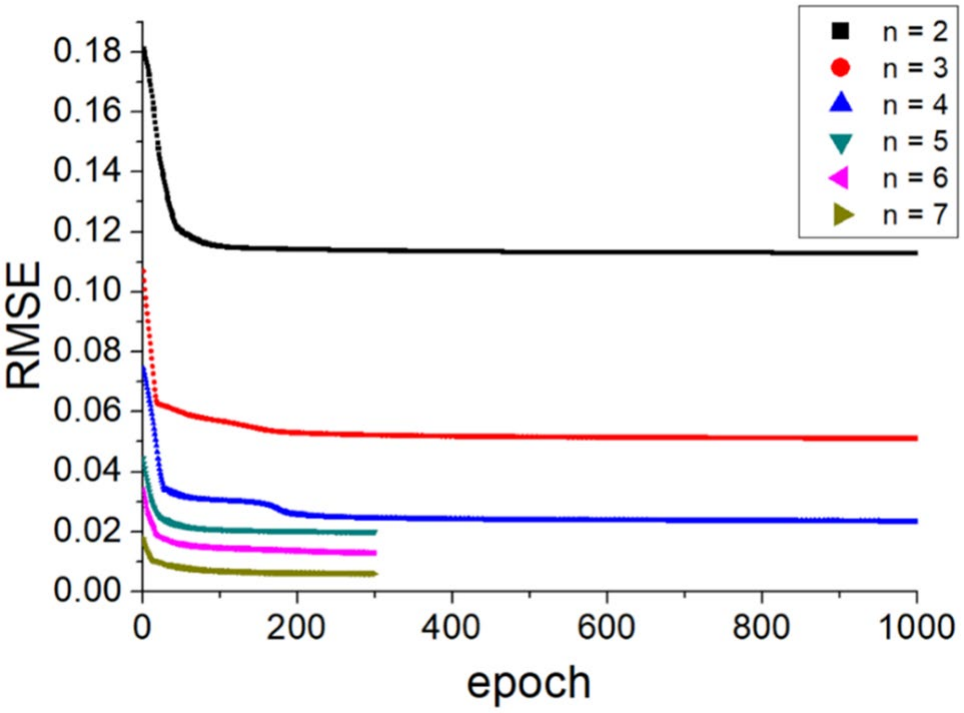
Convergence of the ANFIS model with respect to the number of epochs. The number of epochs reached 300, the degree of convergence of the model was satisfactory.

Figure 6 shows the convergence tendencies of the ANFIS model with respect to the number of epochs. n is the number of input membership functions. When the number of input membership functions was two, three, and four, the RMSE values were 0.11, 0.05, and 0.02 after 1000 epochs, respectively. When the number of epochs reached 300, the degree of convergence of the model was satisfactory. When the number of input membership functions was five, six, and seven, the RMSE values were 0.02, 0.01, and 0.005, respectively.

**Figure 7:**
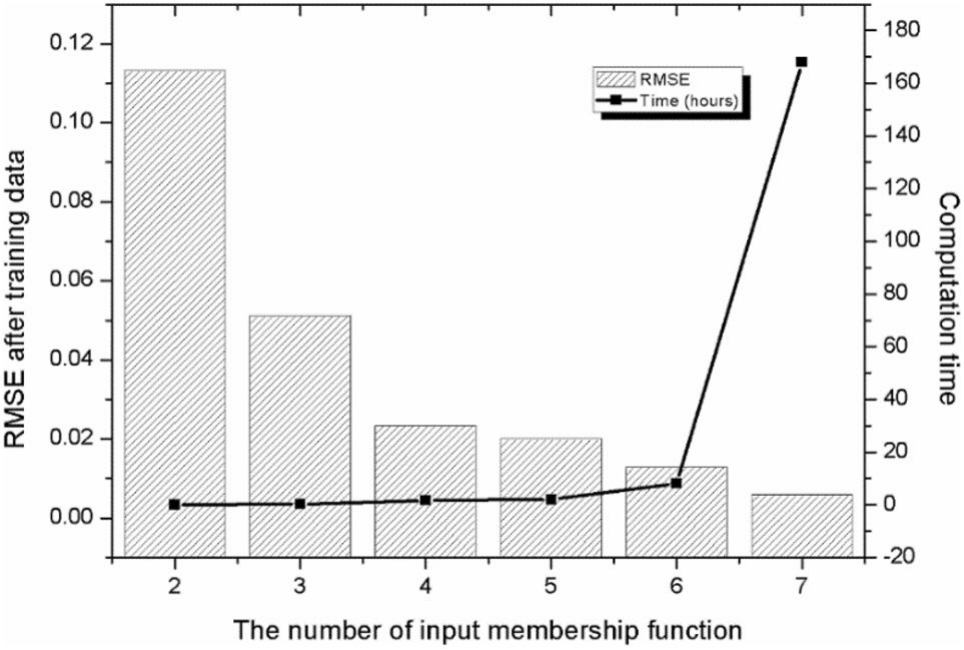
Root mean square error (RMSE) and computation time of the ANFIS model with respect to the number of input membership functions.

Figure 7 shows the RMSE value and computation time of the ANFIS model with respect to the number of input membership functions. The computations were performed on a computer with an Intel Core i5-8400 CPU 2.80 GHz and 16.0 GB of RAM. With 300 epochs and six input membership functions, the ANFIS training took approximately eight hours and satisfied the convergence criterion of RMSE < 0.01. The RMSE value decreased with increasing number of input membership functions. On the other hand, the RMSE value decreased when the number of inputs exceeded seven, but the rate of decrease of the RMSE was very low, and the computational time increased suddenly, which is inefficient. Therefore, six functions and 300 epochs are efficient for convergence.

**Figure 8:**
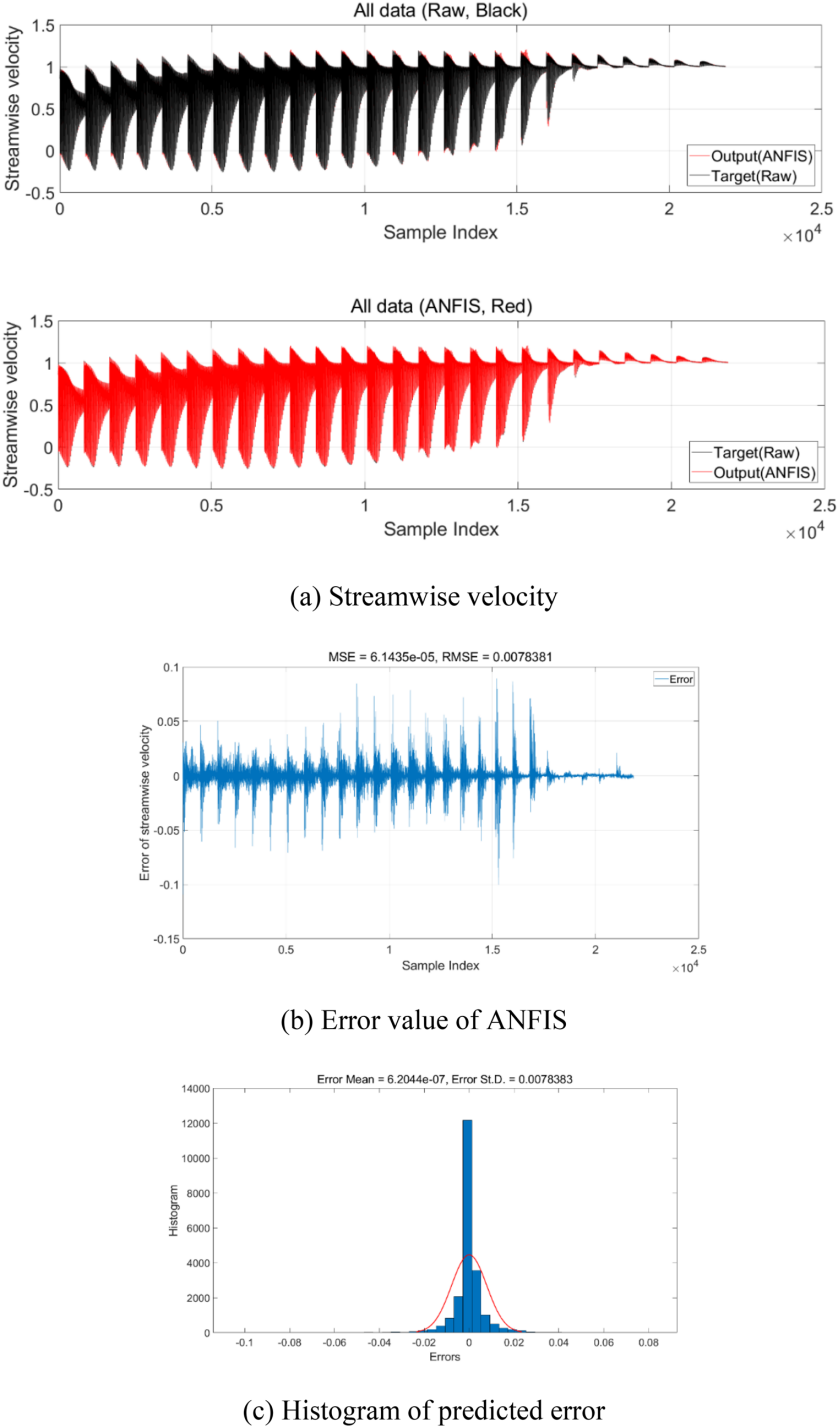
Accuracy of ANFIS model for average streamwise velocity for model 1. (**a**) Comparison of streamwise velocity; (**b**) error of streamwise velocity value; (**c**) histogram of ANFIS error.

Figure 8 shows the ANFIS model’s error of average streamwise velocity compared with measurement data for model 1. Figure 8a shows the comparison with measurement data (target) and ANFIS result (output). For training, the RMSE value of streamwise velocity is 0.0078. This result shows that the error between the predicted data and measurement value is less than 0.78% for streamwise velocity component. Consequently, this shows the high degree of linear dependence R^2^ between the ANFIS and measurement result in the training process. Consequently, the ANFIS model can accurately predict the 4D PTV velocity and aerodynamics with an error of less than 0.78%. This error can be reduced with more input membership function.

## Results and discussion

The ANFIS method can predict the time-resolved three-dimensional velocity field of the side mirror model with less computational time and provide high temporal and spatial resolution. The number of raw data in the x/H, y/H, and z/H mesh coordinates was 28 × 30 × 26 nodes, which have 21,840 data. This coordinate had a step size of 0.05 between the nodes. For ANFIS-based data prediction of 4D PTV, the ANFIS model predicted x/H, y/H, and z/H from 0 to 1.5, − 0.7 to 0.7, and 0 to 1.3, respectively, with step sizes of 0.00625. This means that the spatial resolution of the raw data will increase eight-fold. The total number of nodes is 241 × 225 × 209 nodes (11,333,025 data).

**Figure 9:**
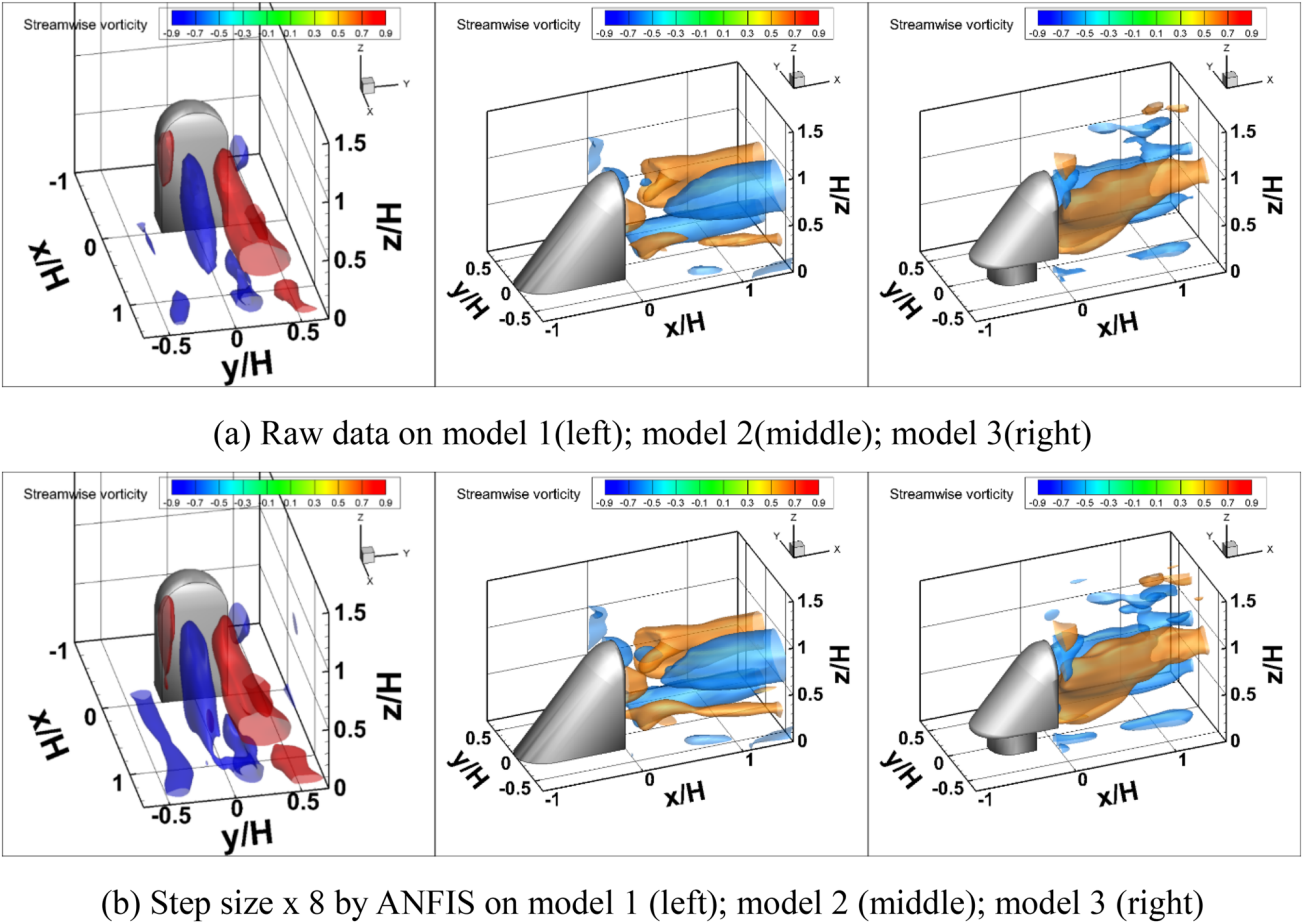
Comparison of the ensemble-averaged 3D streamwise vortex structures between the raw and predicted data. The spatial resolution of the raw data will increase eight-fold by ANFIS.

Figure 9 compares the ensemble-averaged streamwise vortex structures between the raw data and ANFIS data prediction for three different side mirror models. The increase in spatial resolution means that the vorticity, which is a function of the gradient of velocity and space, can be well distinguished. In the case of the ANFIS model, the connection of the horseshoe vortex from the bottom of the model was very clear. On the other hand, in the case of raw data, the horseshoe vortex was broken due to a lack of spatial resolution, as shown in model 1’s result. The sidewall roll-up vortex and trailing vortex pair were also recovered well by ANFIS data prediction. When the flow was developing downstream, the vortex pair inclined towards the ground plane owing to the downwash effect. The streamwise trailing vortex pair showed a dipole distribution, counterclockwise rotation in the left-hand side vortex, and clockwise rotation in the right-hand side vortex. For model 2, the horseshoe vortex disappeared due to the inclination of the model at the front side, but a trailing vortex formed into a dipole formation. In the case of the lower vortex pairs of the dipole trailing vortex, the raw data show that the vortex form is broken. After ANFIS data prediction, this vortex was recovered by the high spatial resolution. For model 3, the horseshoe vortex that occurs at the base of the model is clearer. A horseshoe vortex with small size and magnitude occurs because the base of the model has the same shape as the reference model.

**Figure 10:**
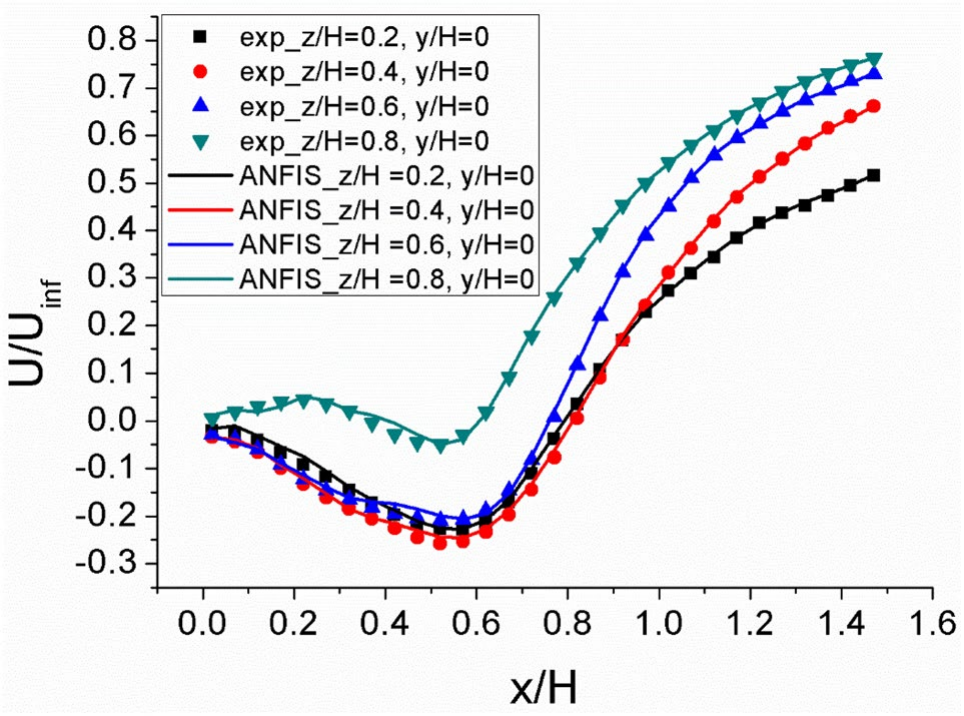
A comparison of normalized average streamwise velocity for experimental result and ANFIS data prediction of model 1 along the x/H at z/H = 0.2 ~ 0.8, y/H = 0.

Figure 10 shows the comparison of normalized average streamwise velocity for experimental result and ANFIS data prediction of model 1. This result shows the predicted and measured streamwise velocity at the x–y plane for different z position. According to the line data, the ANFIS data predictions are in good agreement with the measurement data for all the domain, which is almost same with the PTV results. In comparison to the experimental data, the ANFIS data prediction slightly poor predicts the streamwise velocity in recirculation region. This is because the absolute velocity magnitude in recirculation region is very low compared with another region. It is possible to enhance this poor prediction, different ANFIS setting parameters or data filtering are required, especially the number of input membership function.

The average velocity fields cannot resolve relatively small vortex structures as well as the evolution of the vortex structures. To take more advantages of the ANFIS data prediction, the instantaneous velocity field of the side mirror model 1 was used for data prediction.

**Figure 11:**
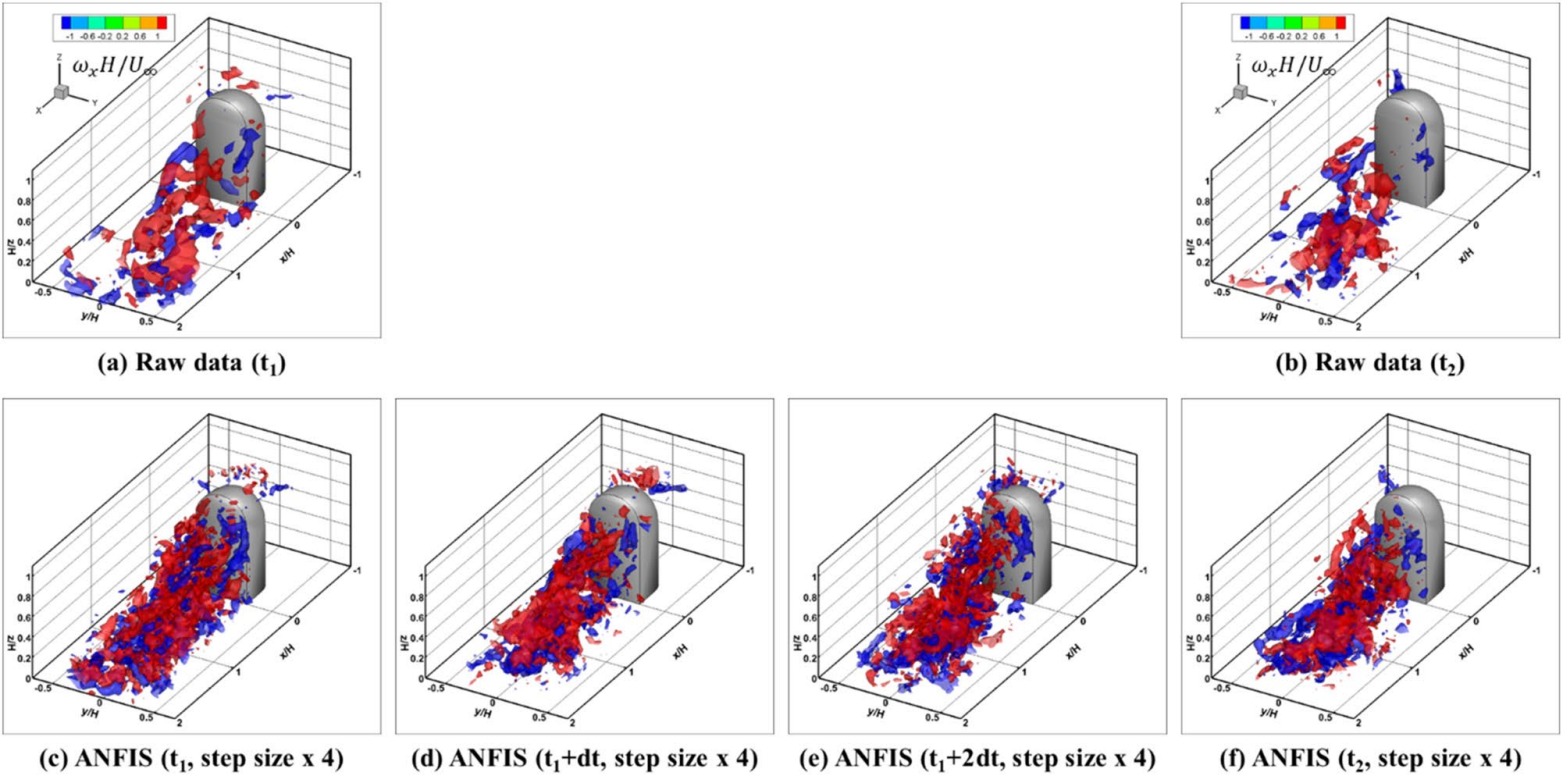
Comparison of the instantaneous 3D streamwise vortex structures between the raw and predicted data. (**a**,**b**) Raw data at t1, t2; (**c**,**f**) spatial enhancement by ANFIS at t1, t2; (**d**,**e**) spatio-temporal enhancement by ANFIS.

Figure 11 presents the instantaneous streamwise vortex structures in the wake of the side mirror model 1. Compared to the ensemble-averaged results (Fig. 9a,b), the effects of data prediction were certainly apparent. The AI-based data prediction results (Fig. 11c,f) showed much smaller vortex structures as a result of the four-fold increase in the spatial resolution. Moreover, the four-times higher temporal resolution of the ANFIS model revealed much more small-scale streamwise vortical structures than those from only an enhancement of the spatial resolution. Compared to the average streamwise vortex structure, the instantaneous vorticity field was not a symmetrical feature. Clusters of the vortex structure rotating clockwise or counterclockwise were inclined to the bottom with downwash flow. During the measurement period of Fig. 11c–f, the vortex structures rotating clockwise were above the vortex structures rotating counterclockwise, but at other times it could be the opposite considering that the time-averaged streamwise trailing vortex structure is symmetrical. These data prediction results provide a better understanding of the small turbulence structures and allow for more in-depth analysis by recovering the missed data due to the resolution limit of the experiment.

**Figure 12:**
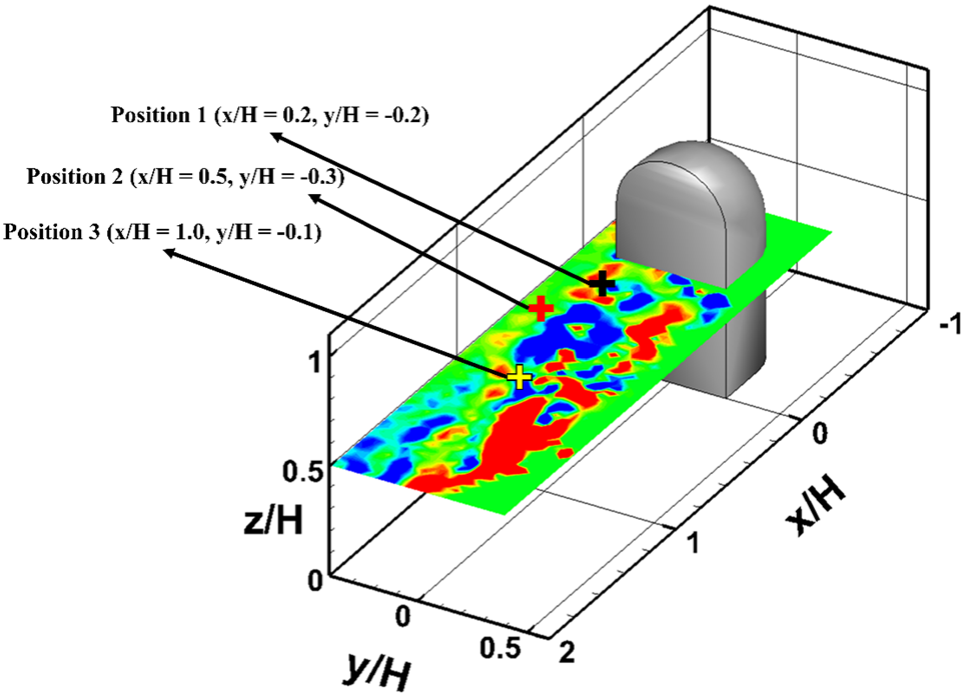
Measurement positions at z/H = 0.5. P1 is in the recirculation zone; P2 is located at the wake shear layer region; P3 is in the area where the trailing vortex appeared near the central region of the wake after passing the recirculation zone downstream of the model.

From the ANFIS model with improved temporal and spatial resolution, it was possible to extract the instantaneous velocity and pressure fluctuations at a specific location in the flow field. After obtaining the power and noise level spectrum from the time series data, it was possible to obtain the shedding frequency of the vortex from the side mirror model and identify the noise source from the fluctuation of the flow pressure. Figure 12 shows three locations where the velocity and pressure are extracted from the horizontal plane at z/H = 0.5 for model 1. Position 1 (P1) is in the recirculation zone (x/H = 0.2, y/H = − 0.2), where backflow occurs behind the model. Position 2 (P2) is located at the wake shear layer region (x/H = 0.5, y/H = − 0.3) with a high streamwise velocity. Position 3 (P3) is in the area where the trailing vortex appeared near the central region (x/H = 1.0, y/H = − 0.1) of the wake after passing the recirculation zone downstream of the model.

**Figure 13:**
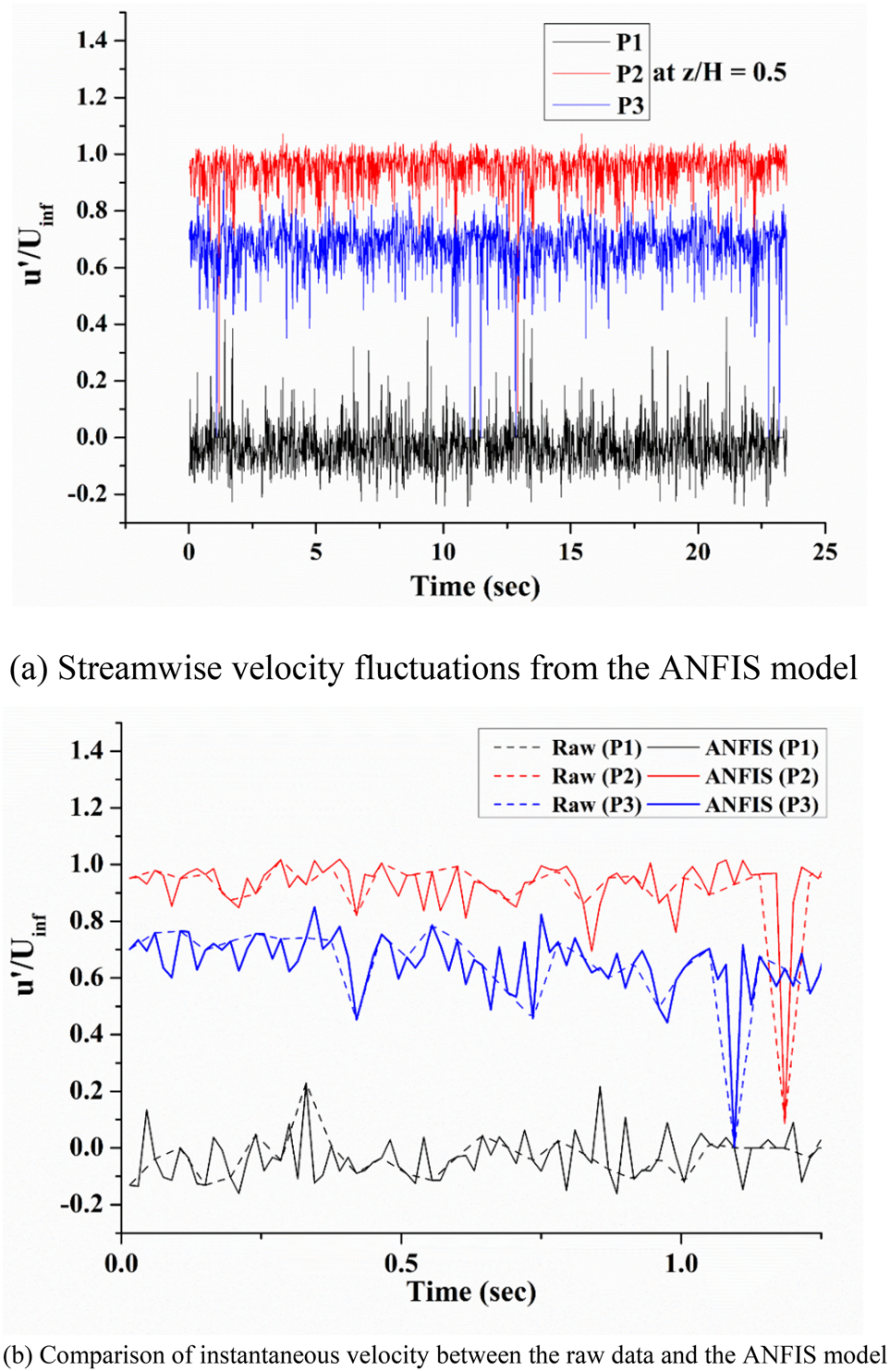
Comparison of the instantaneous streamwise velocity fluctuations between the raw and predicted data taken at P1, P2, and P3 in the horizontal plane at z/H = 0.5 for model 1.

Figure 13a shows the extracted streamwise velocity fluctuations from the ANFIS model at three different positions on model 1 for the entire measurement time, 22 s. The velocity magnitudes were mostly negative at P1 because the location is within the recirculating zone. The velocity fluctuation at P2 had the highest magnitude because the location is just outside of the recirculation zone, where the separated shear layer exists. The time series of the streamwise velocity at P3 had a lower velocity magnitude than that of P2. On the other hand, the turbulent intensity of P3 was higher than that of P2. The position was located in the trailing vortex region, which is inside the wake flow. Figure 13b compares the instantaneous velocity extracted at three different points from the raw velocity data and the ANFIS model with a four-fold higher temporal resolution than the raw data. For a better comparison, only 1.5 s were selected. The overall change was consistent with each other, but the ANFIS model resolved more fluctuations. Every fourth ANFIS result coincided with the raw data because raw data was used as the ground truth in ANFIS learning.

The dominant characteristics of the external flow over a bluff body were vortex shedding, and flow-induced noise is closely related to this phenomenon. The shape of the side mirror model was similar to a half-cylinder and vortex shedding occurred behind the model. Fast Fourier Transform (FFT) analysis was performed with the velocity signals of Fig. 13a.

**Figure 14:**
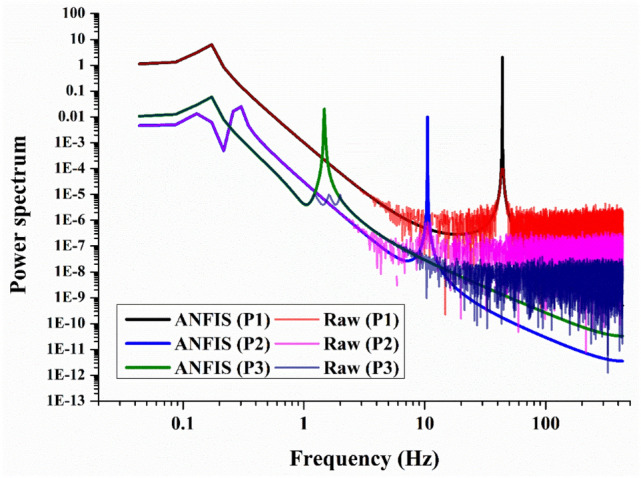
Comparison of the power spectrum from the instantaneous streamwise velocity fluctuation between the raw and predicted data taken at P1, P2, and P3 in the horizontal plane at z/H = 0.5 for model 1.

Figure 14 shows the power spectra of the streamwise velocity fluctuations extracted at the three points. Because the power spectrum was obtained only with the fluctuation component of the velocity, the highest power value came out from position P1, where the fluctuation was the largest, and in the order of P3 and P2. In Fig. 14, the power spectra obtained using the raw velocity data and the instantaneous velocity extracted from the ANFIS model were compared. Both coincided with each other in the low-frequency range below 3 Hz, but there were significant differences in the high-frequency range above 10 Hz. The power spectrum obtained using the raw data revealed a noisy spectrum with the same mean value above 10 Hz. Because the sampling rate of raw data was 289 Hz, the spectrum above 145 Hz was meaningless using the Nyquist sampling criteria, and high power at a lower frequency was derived from aliasing. On the other hand, the ANFIS model had a resolution of 867 Hz. Therefore, it showed a very clean spectrum in the frequency range below 400 Hz.

The instantaneous velocity field obtained as a result of the AI-based data prediction showed the vortex shedding frequency as a very clear peak. The power spectrum tended to decrease with the power-law as it goes into higher frequencies. Interestingly, the peak frequency of the spectrum obtained at each point was different because the structure of the dominant vortex in each position was different. The most prominent frequency peak was 50 Hz measured at the P1 position. This position is closely related to the vortex shedding that occurs on the side of the semi-cylindrical side mirror model. In the P2 position, periodic vortex shedding of approximately 10 Hz occurred. This location was related to the abnormality of the trailing vortex structure. In the spectrum at the P3 position, a low-frequency peak of 1.5 Hz, which was not found in the raw data, appeared in the ANFIS model results. Because the P3 position becomes the point where the recirculating zone ends, it was assumed that it would be related to the slow meandering phenomenon of the separation bubble.

The pressure fluctuation was quantified to compare the noise of the mirror models at P1. Instantaneous pressure fields were deduced by solving a Poisson equation based on the 4D PTV data. The instantaneous pressure extracted from the raw data and the ANFIS model with a four-fold higher temporal resolution. The instantaneous pressure data were converted to sound pressure levels using the following equation and a frequency analysis:

$$ {\text{SPL}} = 20{ } \times {\text{ log}}10\left( {\frac{P}{{P_{ref} }}} \right) $$

P is the instantaneous pressure data, and P_ref_ is the reference sound pressure (20 × 10^–6^ pa was used for sound pressure in air).

**Figure 15:**
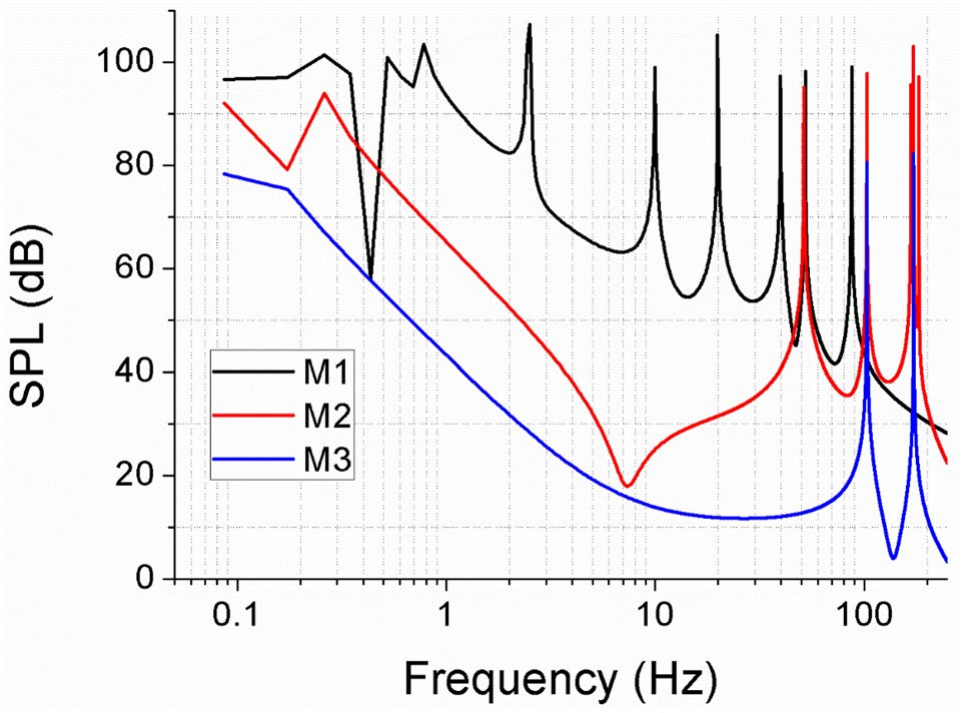
Comparison of predicted sound pressure level at P1 for three different side mirror models.

Figure 15 shows a comparison of the sound pressure level for different side mirror models. The magnitude of the noise level was highest for model 1, followed by model 2 and model 3 at the same position. The peak of model 1 was dominant at 10–100 Hz in the low frequency band. This region has strong air resonance, and most of the noise felt by humans is in this area. At model 1, the peak frequencies were found at 10, 20, 40, 80, and 120 Hz, which are the harmonics based on 10 Hz. At model 2, the peak sound frequencies were observed at 50, 100, and 200 Hz, which are the harmonics of vortex shedding frequency, 50 Hz. At model 3, peaks appeared at 100 and 200 Hz, the same as model 2. Models 2 and 3 have peaks in the mid frequency band but not the low frequency bands, which affects noise.

## Conclusions

An AI-based data prediction technique was developed using 4D robotic PTV measurement for fluid dynamics. By learning 3D or 4D flow patterns through AI, generalization ability of the model was obtained, and error and noise were reduced because statistical data were learned based on a neural network algorithm. In addition, 4D flow measurements of side mirror models were performed to experimentally investigate the 3D flow characteristics. In the case of model 1, the formation of a horseshoe vortex from the bottom of the model was very clearly observed. In the case of model 2, the horseshoe vortex disappeared due to the inclination of the model at the front side, but a trailing vortex formed into a dipole formation. In the case of the lower vortex pairs of the dipole trailing vortexes, the raw data showed that the vortex form is broken. After the ANFIS data prediction, the vortex was recovered by the high spatial resolution. In the case of model 3, a horseshoe vortex occurred at the base of the model and was observed more clearly. For the instantaneous result, compared with raw data, the data prediction results showed a small vortex structure as a result of increasing the spatial resolution. These data prediction results provide a better understanding of the small turbulence structures and allow for more in-depth analysis by recovering missed data. The instantaneous pressure fields were deduced by solving a Poisson equation based on the 4D PIV data and the ANFIS method. The magnitude of the noise level was highest for model 1, and the peak was dominant at 10–100 Hz in the low frequency band, where humans feel noise. Models 2 and 3 had peaks in the mid-frequency band and not the low frequency band. The ANFIS model could help with numerical and experimental methods to optimize case studies without doing experiments. This method could also enable mesh refinement with low computational time.

## References

[1] Yu, C. Automotive wind noise prediction using deterministic aero-vibro-acoustics method. in *23rd AIAA/CEAS Aeroacoustics Conference* 3206 (2017).

[2] Yao HD, Davidson L (2018). Generation of interior cavity noise due to window vibration excited by turbulent flows past a generic side-view mirror. Phys. Fluids.

[3] Wang Y, Gu Z, Li W, Lin X (2010). Evaluation of aerodynamic noise generation by a generic side mirror. World Acad. Sci. Eng. Technol..

[4] Wang, Q., Chen, X., Zhang, Y., & Meng, W. *Unsteady Flow Control and Wind Noise Reduction of Side-View Mirror* (No. 2018-01-0744). SAE Technical Paper (2018).

[5] Wan J, Ma L (2017). Numerical investigation and experimental test on aerodynamic noises of the bionic rear view mirror in vehicles. J. Vibroeng..

[6] Vanherpe, F., Baresh, D., Lafon, P., & Bordji, M. Wavenumber-frequency analysis of the wall pressure fluctuations in the wake of a car side mirror. in *17th AIAA/CEAS Aeroacoustics Conference (32nd AIAA Aeroacoustics Conference)* 2936 (2011).

[7] Kim, J. H., Han, Y. O., Lee, M. H., Hwang, I. H., & Jung, U. H. Surface flow and wake structure of a rear view mirror of the passenger car. in *Proceedings of the Bluff Bodies Aerodynamics & Applications (BBAA) VI*, Milan, Italy 20–24 (2008).

[8] Kharazi, A., Duell, E., Kimbrell, A., & Boh, A. *Prediction of Flow-Induced Vibration of Vehicle Side-View Mirrors by CFD Simulation* (No. 2015-01-1558). SAE Technical Paper (2015).

[9] Rinoshika A, Watanabe S (2010). Orthogonal wavelet decomposition of turbulent structures behind a vehicle external mirror. Exp. Therm. Fluid Sci..

[10] Khalighi, B. & Lee, R. PIV velocity and pressure measurements of the unsteady flow field behind two automobile outside rear view mirrors. *WSAES Trans. Fluid Mech.* **6**(2), 102–112 (2011).

[11] Van Oudheusden BW (2013). PIV-based pressure measurement. Meas. Sci. Technol..

[12] Ebbers T, Farnebäck G (2009). Improving computation of cardiovascular relative pressure fields from velocity MRI. J. Magn. Reson. Imaging.

[13] Pröbsting S, Scarano F, Bernardini M, Pirozzoli S (2013). On the estimation of wall pressure coherence using time-resolved tomographic PIV. Exp. Fluids.

[14] Schanz D, Gesemann S, Schröder A (2016). Shake-The-Box: Lagrangian particle tracking at high particle image densities. Exp. Fluids.

[15] Kim D, Kim M, Saredi E, Scarano F, Kim KC (2020). Robotic PTV study of the flow around automotive side-view mirror models. Exp. Therm. Fluid Sci..

[16] Brunton SL, Noack BR, Koumoutsakos P (2020). Machine learning for fluid mechanics. Annu. Rev. Fluid Mech..

[17] Akdemir, B., Doğan, S., Aksoy, M. H., Canli, E., & Özgören, M. Artificial frame filling using adaptive neural fuzzy inference system for particle image velocimetry dataset. in *Sixth International Conference on Graphic and Image Processing (ICGIP 2014)*, Vol. 9443 94431R. International Society for Optics and Photonics (2015).

[18] Gamahara M, Hattori Y (2017). Searching for turbulence models by artificial neural network. Phys. Rev. Fluids.

[19] Kutz JN (2017). Deep learning in fluid dynamics. J. Fluid Mech..

[20] Lee Y, Yang H, Yin Z (2017). PIV-DCNN: Cascaded deep convolutional neural networks for particle image velocimetry. Exp. Fluids.

[21] Ling J, Kurzawski A, Templeton J (2016). Reynolds averaged turbulence modelling using deep neural networks with embedded invariance. J. Fluid Mech..

[22] Newby JM, Schaefer AM, Lee PT, Forest MG, Lai SK (2018). Convolutional neural networks automate detection for tracking of submicron-scale particles in 2D and 3D. Proc. Natl. Acad. Sci..

[23] Rabault J, Kolaas J, Jensen A (2017). Performing particle image velocimetry using artificial neural networks: A proof-of-concept. Meas. Sci. Technol..

[24] Raissi M, Wang Z, Triantafyllou MS, Karniadakis GE (2019). Deep learning of vortex-induced vibrations. J. Fluid Mech..

[25] Strofer CM, Wu JL, Xiao H, Paterson E (2019). Data-driven, physics-based feature extraction from fluid flow fields using convolutional neural networks. Commun. Comput. Phys..

[26] Yan Y, Safdari A, Kim KC (2020). Visualization of nanofluid flow field by adaptive-network-based fuzzy inference system (ANFIS) with cubic interpolation particle approach. J. Vis..

[27] Xu P, Babanezhad M, Yarmand H, Marjani A (2020). Flow visualization and analysis of thermal distribution for the nanofluid by the integration of fuzzy c-means clustering ANFIS structure and CFD methods. J. Vis..

[28] Choi H, Jeon WP, Kim J (2008). Control of flow over a bluff body. Annu. Rev. Fluid Mech..

[29] Siegel L, Ehrenfried K, Wagner C, Mulleners K, Henning A (2018). Cross-correlation analysis of synchronized PIV and microphone measurements of an oscillating airfoil. J. Vis..

[30] Kim, D. 4D Lagrangian robotic PTV measurement and AI based data assimilation: Flow characteristics of side mirror models. PhD Thesis, Pusan National University (2019).

[31] Jux, C. Robotic volumetric particle tracking velocimetry by coaxial imaging and illumination. PhD Thesis, TU Delft (2017)

[32] Soloff SM, Adrian RJ, Liu ZC (1997). Distortion compensation for generalized stereoscopic particle image velocimetry. Meas. Sci. Technol..

[33] Wieneke B (2008). Volume self-calibration for 3D particle image velocimetry. Exp. Fluids.

[34] Schanz D, Gesemann S, Schröder A, Wieneke B, Novara M (2013). Non-uniform optical transfer functions in particle imaging: Calibration and application to tomographic reconstruction. Meas. Sci. Technol..

[35] Sciacchitano A, Scarano F (2014). Elimination of PIV light reflections via a temporal high pass filter. Meas. Sci. Technol..

